# A Systematic Review of Deep Learning Techniques for Tuberculosis Detection From Chest Radiograph

**DOI:** 10.3389/fmed.2022.830515

**Published:** 2022-03-10

**Authors:** Mustapha Oloko-Oba, Serestina Viriri

**Affiliations:** Computer Science Discipline, School of Mathematics, Statistics and Computer Science, University of KwaZulu-Natal, Durban, South Africa

**Keywords:** tuberculosis, chest radiograph, computer-aided diagnosis, deep learning, systematic review

## Abstract

The high mortality rate in Tuberculosis (TB) burden regions has increased significantly in the last decades. Despite the possibility of treatment for TB, high burden regions still suffer inadequate screening tools, which result in diagnostic delay and misdiagnosis. These challenges have led to the development of Computer-Aided Diagnostic (CAD) system to detect TB automatically. There are several ways of screening for TB, but Chest X-Ray (CXR) is more prominent and recommended due to its high sensitivity in detecting lung abnormalities. This paper presents the results of a systematic review based on PRISMA procedures that investigate state-of-the-art Deep Learning techniques for screening pulmonary abnormalities related to TB. The systematic review was conducted using an extensive selection of scientific databases as reference sources that grant access to distinctive articles in the field. Four scientific databases were searched to retrieve related articles. Inclusion and exclusion criteria were defined and applied to each article to determine those included in the study. Out of the 489 articles retrieved, 62 were included. Based on the findings in this review, we conclude that CAD systems are promising in tackling the challenges of the TB epidemic and made recommendations for improvement in future studies.

## Introduction

Tuberculosis (TB) is ranked among the leading causes of death. About 10 million persons fell ill globally from TB infections in 2019 (1). TB is triggered by the Mycobacterium bacteria that usually affect the lungs (pulmonary) but sometimes affect other parts of the body (extrapulmonary) (2). Many TB patients lose their lives yearly due to diagnostic delay, misdiagnosis, and lack of appropriate treatments (3, 4). Although TB is a global challenge, the mortality rate is more prevalent in low and middle-income nations (5).

TB is certainly treatable if diagnosed early for appropriate treatment. Early diagnosis is essential for successful treatment, preventing further spread, and significantly reducing the mortality rate in line with the World Health Organization (WHO) End TB Strategy (1). The gold standard for TB screening is Sputum culture. However, posterior-anterior chest radiographs (CXR) are an effective technique with low-cost and moderately low radiation doses for screening lung abnormalities to achieve prompt results (6). CXR has been adequately employed in developed countries to analyze individuals exhibiting active TB symptoms. At the same time, its application is limited in developing countries where TB is most prevalent (7, 8). High TB burden regions lack the skilled and radiological expertise required to interpret CXR images adequately (9, 10).

In the last decades, several efforts have been made using Artificial Intelligence (AI) to develop a Computer-Aided Detection (CAD) system to advance automatic object/image recognition tasks and overcome the challenges of a skilled workforce. Machine Learning (ML) and Deep Learning (DL) are the predominant AI techniques employed to develop CAD systems for analyzing CXR images. Both techniques have had a significant impact, but the DL approach, such as Convolutional Neural Network (CNN), has become more prominent for analyzing different pulmonary abnormalities in the medical domain, most importantly in diagnosing TB. The application of an efficient classification tool is vital for improving the quality of diagnosis while reducing the time taken to analyze a large volume of CXRs (11). This endeavor is to achieve the global decline in TB incidence to about 5% annually compared to the current 2% yearly as part of the World Health Organization strategy to end TB (1).

The contribution of this systematic review is to present an extensive summary of the various state-of-the-art CAD system proposed in the literature for the classification of TB. Ultimately, only the CAD system developed using Deep Learning models is considered in this study detailing the diagnostic accuracy between 2017 and 2021. The rest of the paper is structured as follows: section Methodology presents the study methodology. The results are presented in section Results. Section Discussion presents the discussion, while the conclusion is expressed in section Conclusion and Recommendations.

## Methodology

**Figure 1:**
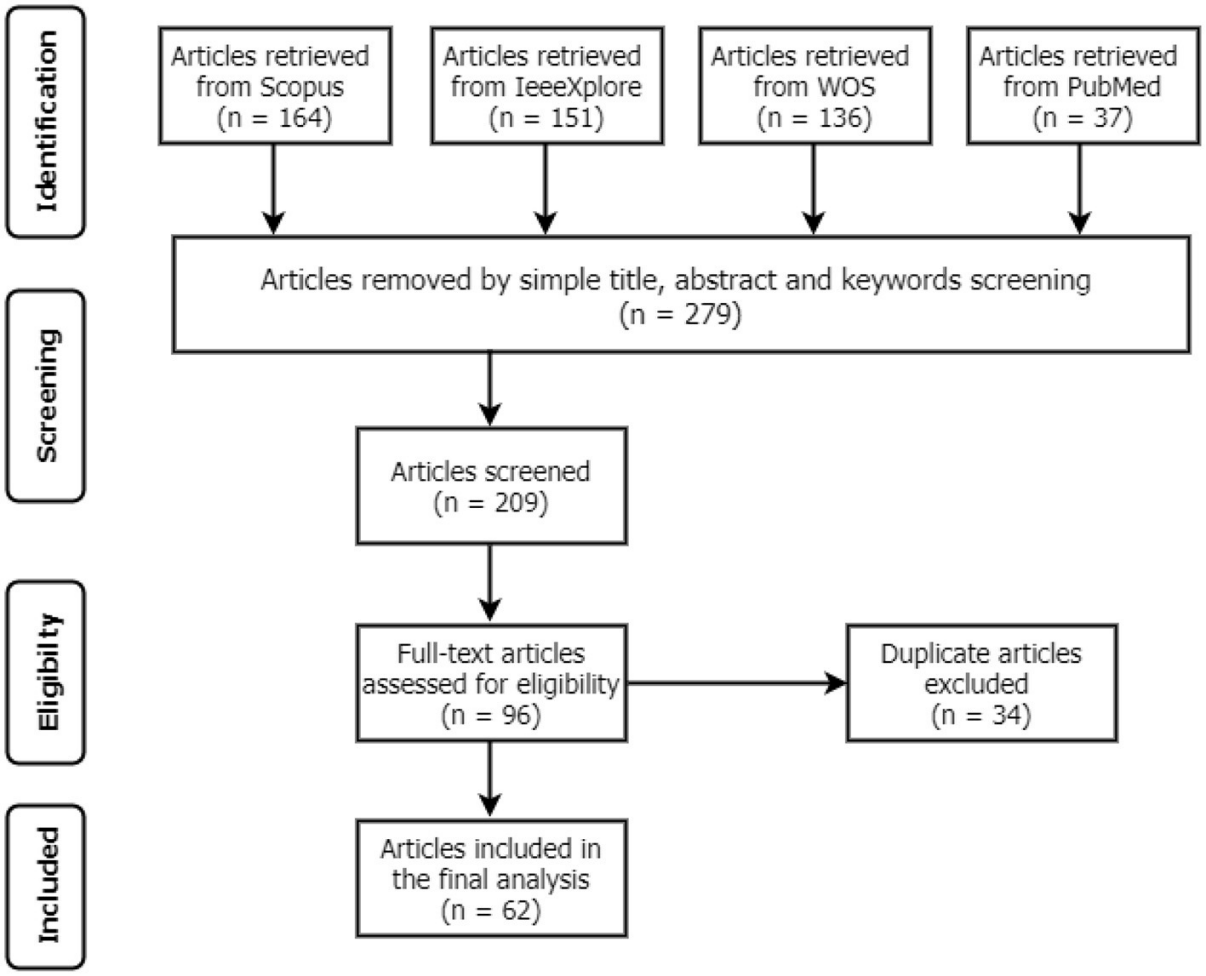
The PRISMA structure for the study selection process.

This systematic review aims to establish various CAD systems related to Tuberculosis diagnosis from CXR using DL techniques. The study followed the Preferred Reporting Items for Systematic Reviews and Meta-Analyses (PRISMA) procedures (12) to identify the standards for inclusion and exclusion, as shown in Figure 1. These standards were formulated based on the present study objectives and the research questions. All articles that satisfied the following conditions were included and excluded if otherwise:

- Articles that considered only pulmonary tuberculosis disease.
- Employed at least one deep learning technique as a classifier
- CXR is the only medical imagine for screening tuberculosis.
- Articles published between January 2017 and September 2021.
- Articles are entirely written in English.
- Articles must be full text. All others, such as abstract, preprints are excluded.

### Search Strategy

The search strategy was developed to identify relevant published articles in Scopus (https://www.scopus.com/), IEEEXplore (https://ieeexplore.ieee.org/), Web of Science (www.webofscience.com), and PubMed (https://www.ncbi.nlm.nih.gov/pubmed/). Searches were performed across the four databases using the following search keywords: “tuberculosis,” “chest x-ray,” “classification,” “artificial intelligence,” “computer-aided diagnosis,” “deep learning.” These keywords were used to form Boolean search strings according to searching standards on different databases. The configuration of keywords used to retrieve all relevant articles from each database search engine is presented in Table 1.

**Table 1:** Construction of search keywords.

**Databases**	**Search keywords**
Scopus	(TITLE-ABS-KEY (“Tuberculosis” AND “Chest X-Ray”) AND TITLE-ABS-KEY (“Deep learning” OR “Machine learning” OR “Artificial Intelligence” OR “Classification”)
IEEEXplore	(Tuberculosis) AND (“Chest X-Ray”) AND (“Deep learning” OR “Machine learning” OR “classification” OR “artificial intelligence”) AND (“CAD” OR “computer-aided detection”)
Web of Science	((Tuberculosis AND Chest x-ray) AND (“Machine learning” OR “Deep learning” OR “Artificial intelligence”) AND (“classification” OR “classify”) AND (“computer-aided diagnosis” OR “CAD”))
PubMed	(“Tuberculosis”) AND (“chest x-ray”) AND (“deep learning” OR “convolutional neural network”) AND (“classify” or “classification”) OR (“computer-aided diagnosis” OR “computer-aided detection” OR “CAD”)

### Study Selection

**Figure 2:**
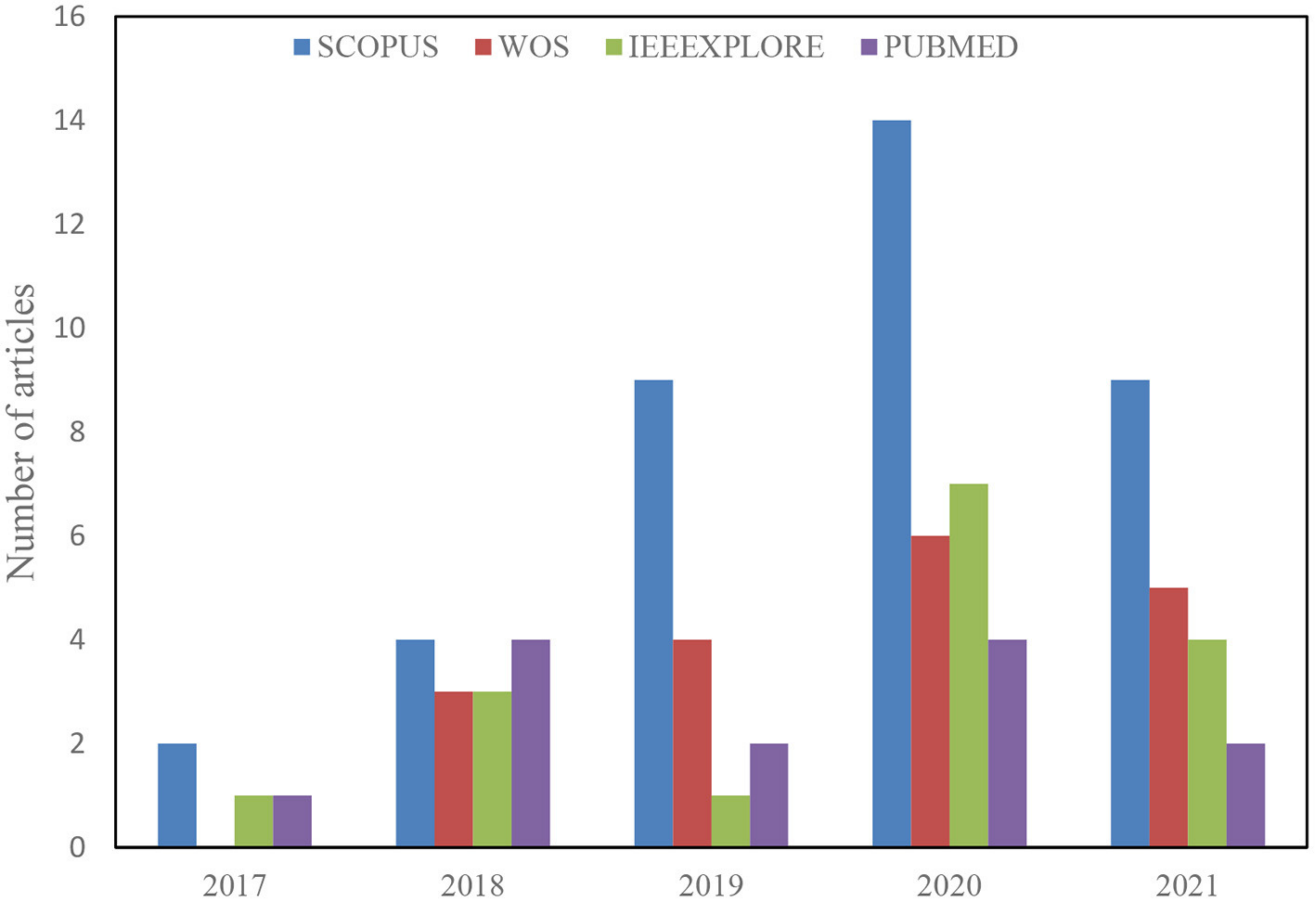
Articles selection by database and year.

A total of 488 articles were retrieved from the initial search on all the databases. The next step then scans the article topics and keywords to identify highly related papers from the irrelevant ones. This step is followed by the overview reading of the abstract, methods, and Conclusion to further screen for relevant papers for full-text reading and better understanding. Thus, 209 articles were considered for the title and abstract screening, out of which 96 papers were found suitable for full-text reading, and a total of 62 were finally included in the analysis after removing 34 duplicates. The detailed structure for the study selection is presented in Figures 1, 2 shows the numbers of articles included per year and databases. It is necessary to note that some relevant articles might have been unintentionally omitted.

### Data Extraction

The details extracted from each article are presented in Tables 2–5. Each table represents the search results from the Scopus, IEEE Xplore, PubMed, and Web of Science databases. Data extracted include study aim and scope, computational techniques (DL models), CXR datasets, evaluation metrics/results achieved, and publication year. The database search shows that most IEEEXplore articles are conference proceedings and included in this systematic review, along with a few conference articles from springer and ACM. This is due to the articles' quality and contribution to the subject matter.

**Table 2:** Scopus search results.

**References**	**Computational techniques**	**Study aim/scope**	**Datasets**	**Results**	**Year**
Duong et al. (13)	EfficientNet, Vision Transformer	Classification of CXR as normal, pneumonia and TB	Montgomery, Shenzhen, Belarus, RSNA, A COVID-19 CXR	Acc = 97.72%, Auc = 100%	2021
Norval et al. (14)	AlexNet, VGG16, and VGG19	Improve accuracy and Classification of CXR as Has TB and No TB	Montgomery, Shenzhen, NIH	Acc = 89.99%	2021
Rahman et al. (15)	ResNet101, VGG19, and DenseNet201 XGBoost	The study utilizes three Deep CNN models as features extractor then classify using eXtreme Gradient Boosting	Montgomery, Shenzhen, Belarus, RSNA, Private	Acc = 99.92% Auc = 99.93% Pre = 99.85% Sen = 100% Spe = 99.85% F1 = 99.92%	2021
Govindarajan and Swaminathan (16)	ELM, OSELM	Identify and classify TB conditions from healthy subjects in chest radiographs using integrated local feature descriptors and variants of extreme learning machine	Montgomery	Acc = 99.2% Sen = 99.3% Spe = 99.3% Pre = 99.0% F1 = 99.20%	2021
Alawi et al. (17)	CNN	Study proposed automated technique to diagnose TB from CXR	NLM, Belarus, NIAID TB and RSNA	Acc = 98.71% Sen = 98.86% Spe = 98.57%	2021
Khatibi et al. (18)	Complex networks and stacked ensemble (CCNSE), CNN	A multi –instance classification model to detect TB from CXR is proposed	Shenzhen, Montgomery	Acc = 99.26%, Auc = 99.00%	2021
Ayaz et al. (19)	Deep CNN	Present a hybrid method for TB detection	Montgomery, Shenzhen	Auc = 0.99%	2021
Priya and Vimina (20)	VGG-19, RestNet50, DenseNet121, InceptionV3	The study employs transfer learning for TB diagnosis.	Montgomery, Shenzhen	Auc = 0.95%	2021
Dasanayaka and Dissanayake (21)	DC-GAN, VGG16 and InceptionV3	generate, segment, and classify CXR for TB using three deep architectures	Montgomery, Shenzhen	Acc = 97.10% Sen = 97.90% Spe = 96.20%	2021
Msonda et al. (22)	AlexNet, GoogLeNet, ResNet50 and Spatial Pyramid Pooling (SPP)	Integrate SPP with Deep CNN to improve performance for TB detection	Montgomery, Shenzhen, Private (KERH)	Acc = 0.98% Pre = 1.00% Spe = 1.00% F1 = 1.00%	2020
Owais et al. (23)	Fusion-based deep classification network	The study proposes a CAD system for the effective diagnosis of TB and provides visual with descriptive information that is useful to the radiologist	Montgomery, Shenzhen	Acc = 0.928% Auc = 0.965% Pre = 0.937% Recall = 0.921% F1 = 0.929%	2020
Yoo et al. (24)	ResNet18	Classify CXR into Normal, TB and Non-TB	Montgomery, Shenzhen, NIH	Acc = 0.98% Sen = 0.98% Spe = 0.97% Auc = 0.965% Pre = 0.97%	2020
Sathitratanacheewin et al. (25)	DCNN	To examine the generalization of deep CNN models for classification of CXR as normal or abnormal with different manifestations	Shenzhen, NIH (ChestX-ray8)	Acc = 0.985% Sen = 72% Spe = 82%	2020
Sahlol et al. (26)	MobileNet Artificial Ecosystem-based Optimization (AEO)	Classification of CXR to detect TB	Shenzhen, Private	Acc = 94.1%	2020
Das et al. (27)	InceptionV3	Screening CXR for TB abnormalities	Montgomery, Shenzhen	Acc = 91.7% Auc = 0.96% Sen = 0.89% Spe = 0.93% Pre = 0.93%	2020
Rahman et al. (28)	ResNet, ChexNet, InceptionV3, Vgg19, DenseNet201, SqueezeNet, MobileNet, and Ensemble	Automatic detection of TB from the CXR.	NLM, Belarus, NIAID TB, and RSNA	Acc = 98.6% F1 = 98.56% Sen = 98.56% Spe = 98.54% Pre = 98.57%	2020
Munadi et al. (29)	ResNet and EfficientNet	Enhances CXR images for improving TB detection accuracy	Shenzhen	Acc = 91.7% Auc = 0.96%	2020
Oloko-Oba and Viriri (30)	CNN	Detection of TB from CXR and classification as normal and abnormal	Shenzhen	Acc = 87.8%	2020
Xie et al. (31)	Faster RCNN	Detection of multiple categories of TB lesions in CXR	Montgomery, Shenzhen	Acc = 0.926% Auc = 0.977%	2020
Verma et al. (32)	InceptionV3, faster RCNN	Classify CXR as pulmonary TB and Pneumonia	Shenzhen	Acc = 99.01%	2020
Tasci (33)	AlexNet, VGGNet	classifying CXR ROI for TB detection	Montgomery, Shenzhen	Acc = 88.32% Auc = 0.92%	2020
Rajaraman and Antani (34)	Inception-V3, ResNet-V2, VGG-16, Xception, DenseNet-121, Ensemble	Improve state-of-the-art architecture for TB detection from CXR	Shenzhen, RSNA, Indiana	Acc = 0.941% F1 = 0.941% Sen = 0.926% Spe = 1.00% Auc = 0.990%	2020
Abideen et al. (35)	B-CNN	Identification and classification of CXR as TB and Non-TB	Montgomery, Shenzhen	Acc = 96.42%	2020
Hijazi et al. (36)	Ensemble of VGG16 InceptionV3	Detection of TB from CXR	Montgomery, Shenzhen	Acc = 89.77% Sen = 90.91% Spe = 88.64%	2019
Pasa et al. (37)	CNN	Developed a faster TB detection algorithm	Montgomery, Shenzhen, Belarus	Acc = 84.4% Auc = 0.900%	2019
Meraj et al. (38)	VGGNet, RestNet50, GoogLeNet	Detection of TB abnormalities from CXR	Montgomery, Shenzhen	Acc = 86.74% Auc = 92.0%	2019
Ahsan et al. (39)	VGG16	Screening of CXR to identify the presence of TB	Montgomery, Shenzhen	Acc = 81.25%	2019
Nguyen et al. (40)	ResNet-50, VGGNet, DenseNet-121, Inception, ResNet.	Improving detection rate of TB	Montgomery, Shenzhen, NIH-14	Auc = 0.99%	2019
Ho et al. (41)	InceptionResNetV2, ResNet150, DenseNet-121	Classification of CXR as pulmonary TB or healthy.	ChestX-ray14, Shenzhen, Montgomery	Auc = 0.95%	2019
Heo et al. (42)	VGG19, InceptionV3, ResNet-50, DenseNet-121, InceptionResNetV2.	Detection of TB from CXR	Privete (Yonsei University)	Auc = 0.9213% Sen = 0.815% Spe = 0.962%	2019
Hernández et al. (43)	Ensemble of VGG19, InceptionV3, ResNet-50	Automatic classification of CXR for TB detection.	Private	Acc = 0.8642.%	2019
Hijazi et al. (44)	Ensemble of InceptionV3, VGG16	Detection of TB from CXR without segmentation	Shenzhen, Montgomery	Acc = 91.0% Sen = 89.6% Spe = 90.7%	2019
Abbas and Abdelsamea (45)	AlexNet	Classification of CXR as healthy or having TB manifestation	Montgomery	Auc = 0.998% Sen = 0.997% Spe = 0.999%	2018
Karnkawinpong and Limpiyakorn (46)	AlexNet, VGG16, CapsNet	CAD for early diagnosis of TB	Private (Thai), Shenzhen, Montgomery	Acc = 90.79% Sen = 89.07% Spe = 92.50%	2018
Stirenko et al. (47)	DCNN	Prediction of the presence of TB from CXR	Shenzhen	———	2018
Becker et al. (48)	CNN	Detection and classification of different TB pathologies from CXR	Private	Auc = 0.98%	2018
Liu et al. (49)	AlexNet, GoogLeNet	Detection and classification of TB manifestations in CXR images	Peruvian	Acc = 85.68%	2017
Hooda et al. (3)	DCNN	Detect and classify TB from CXR as normal and abnormal	Shenzhen, Montgomery	Acc = 82.09%	2017

**Table 3:** PubMed search results.

**References**	**Computational techniques**	**Study aim/scope**	**Datasets**	**Results**	**Year**
Oloko-Oba and Viriri (50)	Ensemble of VGG-16, ResNet50, Inception V3	Automatic detection of TB from CXR	Shenzhen, Montgomery	Acc = 96.14% Sen = 90.03% Spe = 92.41%	2021
Lee et al. (51)	DLAD	Detection of active TB and classification of relevant abnormalities on CXR	Private	Auc = 0.967% Sen = 0.821% Spe = 0.997%	2021
Zhang et al. (52)	Convolutional Block Attention Module (CBAM)	Classification of TB from CXR	Private	Acc = 87.7% Auc = 94.3% Recall = 89.7% Spe = 85.9.7%	2020
Hwang et al. (53)	DLAD	Developed a Deep Learning-based automatic detection algorithm (DLAD) for active Pulmonary TB on CXR and validate its performance using various datasets compared to physicians' results.	Shenzhen, Montgomery, Private (SNUH)	Auc = 0.977% Sen = 94.3% Spe = 91.1%	2019
Rajpurkar et al. (54)	CNN (CheXNeXt)	To evaluate the effectiveness of CheXNeXt in detecting TB and other abnormalities from CXR	ChestX-ray14	Auc = 0.862% Sen = 0.594% Spe = 0.927%	2018
Lakhani and Sundaram (55)	Ensemble of AlexNet, GoogLeNet	Evaluates the efficacy of deep models for detecting TB on CXR	Belarus, Shenzhen, Montgomery	Auc = 0.99% Sen = 97.3% Spe = 94.7%	2017

**Table 4:** IEEE Xplore search results.

**References**	**Computational techniques**	**Study aim/scope**	**Datasets**	**Results**	**Year**
Cao et al. (56)	DenseNet121 VGGNet16, VGGNet19, ResNet152	Evaluates the performance of deep learning for classification of CXR for TB	Shenzhen, Montgomery	Acc = 90.38% F1 = 90.36% Pre = 90.33% Recall = 90.53%	2021
Karaca et al. (57)	VGG16, VGG19, DenseNet121, MobileNet, InceptionV3	Development of a TB detection system	Montgomery	Acc = 98.9% Auc = 1.00%	2021
Saif et al. (58)	DenseNet169, ResNet-50, InceptionV3	Detection of TB from CXR	Shenzhen, Montgomery	Acc = 99.7% Sen = 97.5% Spe = 98.4%	2021
Das et al. (27)	InceptionV3	Screening TB from CXR to eliminate patents diagnosis delay	Shenzhen, Montgomery	Acc = 91.7% Auc = 0.96%	2021
Imam et al. (59)	Modified Inception	They analyzed patients' CXR to determine those infected with TB or not.	Shenzhen, Montgomery	Acc = 91%	2020
Griffin et al. (60)	R-CNN	Location of TB manifestations on CXR	Peruvian	Auc = 0.753% Sen = 0.922% Spe = 0.666%	2020
Rashid et al. (61)	Ensemble of ResNet, Inception-ResNet, DenseNet	Development of a CAD system to classify CXR as normal and infected with TB	Shenzhen	Acc = 90.5%	2018
Abbas and Abdelsamea (45)	AlexNet	Classification of CXR as healthy and unhealthy with TB manifestation	Shenzhen, Montgomery	Auc = 0.998% Sen = 0.999% Spe = 0.997%	2018

**Table 5:** Web of Science search results.

**References**	**Computational techniques**	**Study aim/scope**	**Datasets**	**Results**	**Year**
Fehr et al. (62)	CAD4TBv5	Implement CAD4TB to screen for TB on CXR	Private	Sen = 82.8% Spe = 68.0%	2021
Vats et al. (63)	CNN (iDoc-X)	Diagnosis of TB manifestations from CXR and classify images as TB and Non-TB	Private	Acc = 91.10%	2021
Khatibi et al. (18)	CNNs, complex networks and stacked ensemble	TB recognition from CXR images	Shenzhen, Montgomery	Acc = 99.26% Auc = 99.00%	2021
Rajpurkar et al. (64)	DenseNet121	Development of CheXaid for diagnosing TB	Private	Acc = 0.78% Auc = 0.83%	2020
Grivkov and Smirnov (65)	InceptionV3	Screening of CXR to detect TB pathologist	Shenzhen, Montgomery	Acc = 0.868%	2020
Msonda et al. (22)	AlexNet, GooLeNet, ResNet50, SPP	Integrate SPP with DCNN for the diagnosis of TB on CXR.	Shenzhen, Montgomery, Private (KERH)	Auc = 0.98% Spe = 1.0% Pre = 1.0% F1 = 1.0% Recall = 0.99%	2020
Gozes and Greenspan (66)	DenseNet121	Learned specific features from CXR to detect TB	Chest X-ray14	Auc = 0.965%	2019
Karnkawinpong and Limpiyakorn (67)	AlexNet, VGG-16, CapsNet	Classification of TB from CXR	NLM, Private	Acc = 94.56% Sen = 92.83% Spe = 96.06%	2019
Sivaramakrishnan et al. (68)	AlexNet, VGG16, VGG19, Xception, ResNet-50	evaluate the performance of Deep models toward improving the accuracy of TB screening from CXR	Shenzhen, Montgomery, Private	Acc = 0.855% Auc = 0.956%	2018
Vajda et al. (69)	CNN	Screening CXR to determine which CXR images are normal or abnormal with TB.	Shenzhen, Montgomery	Acc = 97.03% Auc = 0.99%	2018

## Results

This study reviewed computer-aided diagnosis systems articles to detect pulmonary TB from January 2017 until September 2021. It was observed from the articles that the development of CAD follows a standard framework involving four steps as follows: “pre-processing” is the first step which deals with cleaning up the CXR images by eliminating noise and enhancing for clarity. The second step is “segmentation” of a region of interest from the entire image, which is the lung field region in the case of CXR. The third step is the “Feature extraction,” where discriminative features are identified and selected for further analysis in the “classification” step, where the various images are categorized as normal or abnormal (infected) with TB. Several techniques such as handcraft, machine learning, and deep learning have been employed to diagnose TB, but DL has recorded more success in this regard; hence our interest was to analyze the CAD system based on one or more DL techniques as the classifier for TB detection.

The descriptive analysis of the results is presented in Tables 2–5. These tables show the computational technique, study scope, datasets, evaluation criteria, and results. Articles that utilized CXR as the only imaging modality and employed DL as the only computational technique for developing CAD are considered.

### Imaging Modalities

Different screening procedures are used to confirm the presence of TB. Still, Chest Radiograph, otherwise referred to as Chest X-ray (CXR), is a radiograph tool used to detect abnormalities in the lungs and nearby structures. In recent years, CXR has remained a vital method for screening TB and other lung diseases and hence recommended by WHO due to its high sensitivity, wide availability, and relatively less expensive (70, 71).

### Deep Learning Techniques

Many DL techniques have been used for screening, predicting, and diagnosing TB. In most classification algorithms, a dataset is required for training and testing with many samples of inputs and outputs to learn from. Model is developed using the training set to calculate how best to map examples of input data to a specific class label; then, the model validation is accomplished using the test set (72).

In Tables 2–5, only the results obtained from the test set are extracted. Some studies employed more the one DL technique to find the optimal results for diagnosing TB diseases. Only the best results are documented in this study if more than one accuracy is reported in an article.

### Datasets

**Figure 3:**
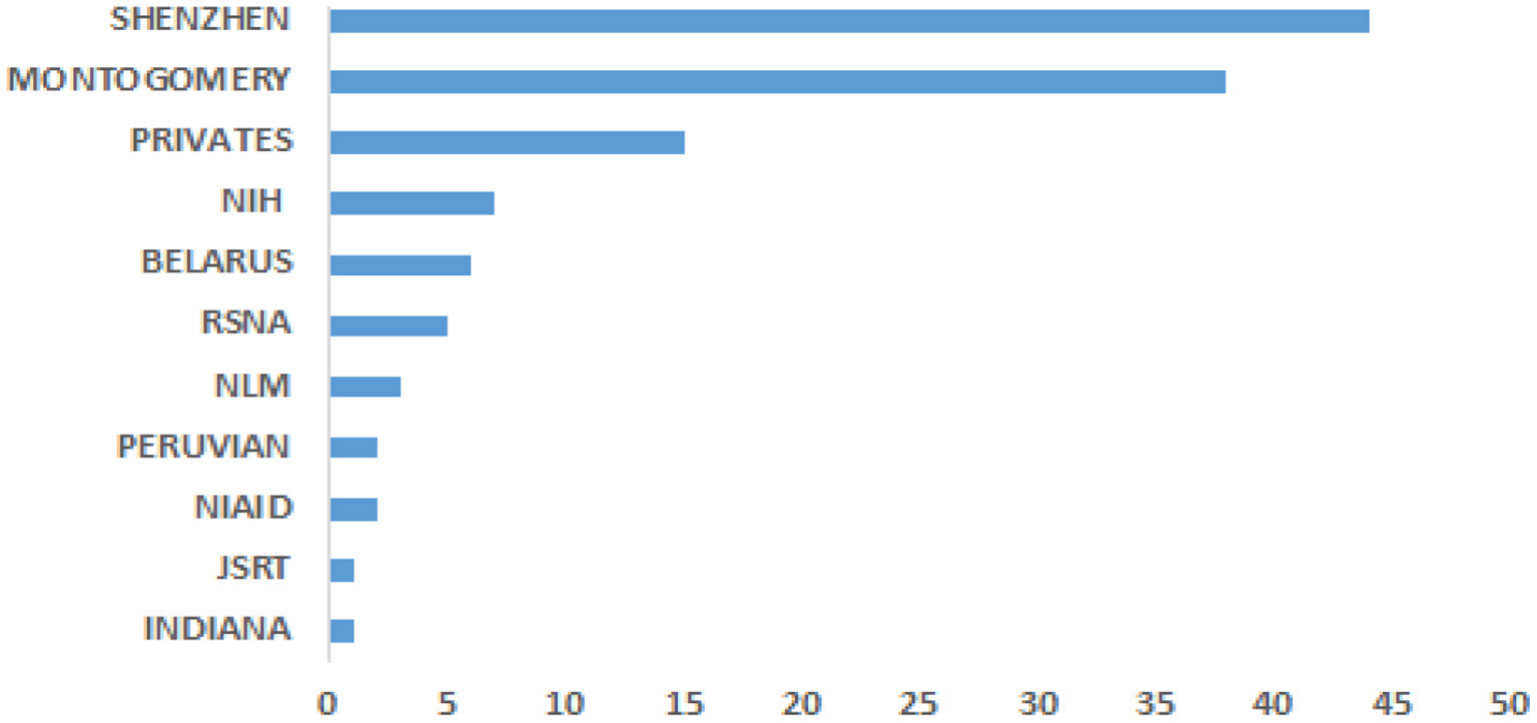
Dataset frequency of usage.

Data is crucial for developing CAD required to solve life-threatening diseases, including TB, as one of the leading causes of worldwide death. The various popular datasets that have been used in developing TB detection algorithms contain de-identified CXR images to protect the privacy of the patients. In other words, the identity of the patients is not disclosed. Most of the datasets are accompanied by radiological interpretation of the observed manifestation that can serve as groundtruth. These datasets are made available to the research communities to foster state-of-the-art research into finding lasting solutions to the early diagnosis of TB manifestations. Some of these public datasets include Montgomery County (73), Shenzhen (73), Peruvian (49), KIT (74), MIMIC-CXR (75), Belarus, NIH (chest x-ray 8, 14) (76), JSRT. Figure 3 shows the datasets frequency of use.

### Evaluation Metrics

Once a model is trained using some training images, the test dataset is then employed to assess the quality of the model. It is evident from the data extraction process that different evaluation metrics exist for assessing model performance. Most of the popular evaluation metrics applied to the development of CAD system includes accuracy, sensitivity, specificity, and AUC. These evaluation metrics are briefly explained as follows:

Accuracy is the rate of the correct samples from the total number of samples examined (77). The equation gives the accuracy:


$$\begin{array}{l} Accuracy\;=\;\frac{TP\;+\;TN}{TP\;+\;FP\;+\;TN\;+\;FN} \end{array}$$

Sensitivity is the proportion of the actual positive samples that are correctly identified as positive (78). This metric is given as:


$$\begin{array}{l} Sensitivity\;=\;\frac{TP}{TP\;+\;FN} \end{array}$$

Specificity is the ratio of the actual negative samples that are correctly identified as negative (78). This metric is given as:


$$\begin{array}{l} Specificity\;=\;\frac{TN}{TN\;+\;FP} \end{array}$$

Precision, otherwise known as a positive predictive value, is the ratio of positive samples that are accurately predicted (77). This is given as:


$$\begin{array}{l} Precision\;=\;\frac{TP}{TP\;+\;FP} \end{array}$$

AUC-ROC Curve: sometimes written as AUROC, specify the rate at which a model distinguishes between classes [84]. It tells the probability of separating negative samples from positive samples. Thresholds are set to determine the ROC curve, which is the plot of True Positive Rate (TPR) against False Positive Rate (FPR) given as:


$$\begin{array}{l} TPR\;=\;Sensitivity=\;\frac{TP}{TP\;+\;FN}\\ FPR\;=\;1\;-\;Specificity=\;\frac{FP}{FP\;+\;TN} \end{array}$$

Where TP, TN, FP, FN, TPR, and FPR are true positive, true negative, false positive, false negative, true positive rate, and false positive rate.

## Discussion

**Figure 4:**
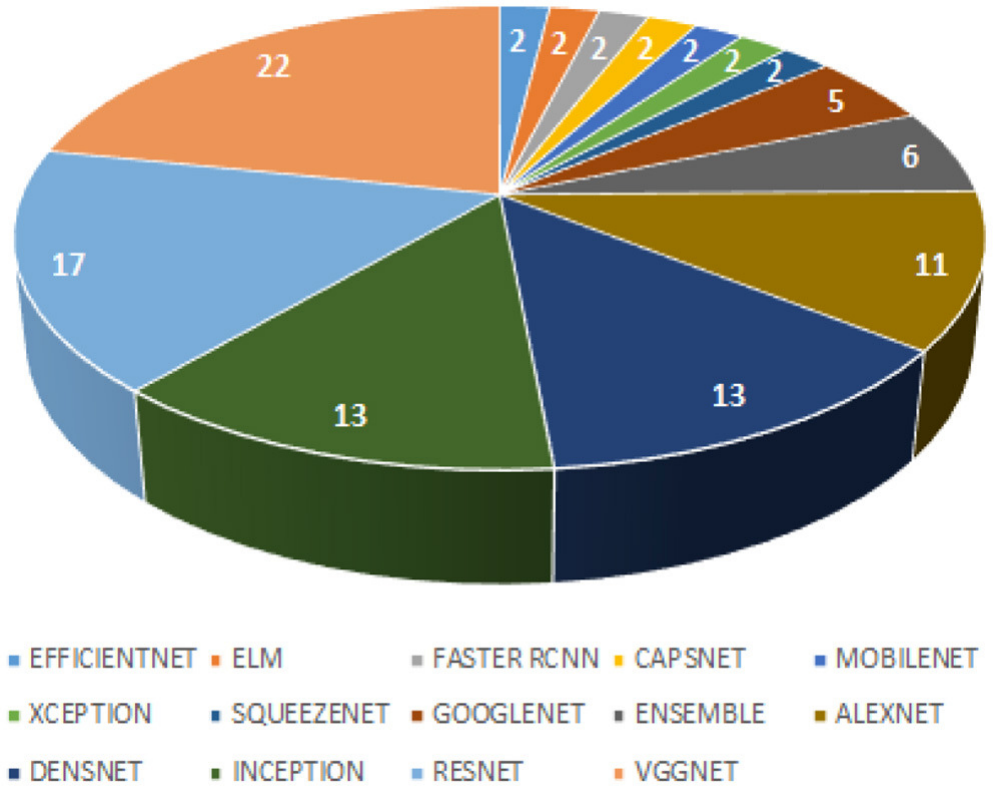
Hierarchical chart of computational techniques according to the frequency of usage.

This systematic review extensively searched the various Deep Learning classifiers for diagnosing TB from CXR. Automatic detection of TB has received mammoth attention in the last decades resulting in many publications with state-of-the-art techniques. Figure 4 presents a hierarchical chart of computational techniques categorized according to the frequency of usage in CAD systems for TB based on the included articles. Despite some good accuracies reported in some studies, we found some limitations in the existing studies concerning methods and reported accuracy, which should be a point of consideration for developing CAD systems in the future. Many studies measured diagnostic accuracy without evaluating the risk of bias emerging from the datasets that were used. It is essential that the accuracy of CAD systems is evaluated using a different set of datasets (CXR images) from the set used for training. In other words, avoid

- Using the same set of CXR images for training and testing.
- Testing with CXR images that were not used for training but originated from the same image subset.
- Using images with class imbalance, and
- Using unannotated CXR images.

Otherwise, the diagnostic accuracy evaluation is likely to be exaggerated and could impact the overall generalization of the system. In general, about 80% of the studies used the same CXR images for training and testing or did not report on it.

## Conclusion and Recommendations

This systematic review intends to inform researchers of the existing Deep Learning classifiers and assist them in developing a CAD system for the efficient diagnosis of TB. Different state-of-the-art Deep Learning techniques that have been used to detect TB have been explored and presented in Tables 2–5. Generally, it is challenging to compare the methods with each other extensively. Factors like the types of datasets used for evaluation, number of image samples used, evaluation metrics approach, and model parameters tunning make the comparison complex.

As evident from the literature, these pre-trained models (VggNet, ResNet, AlexNet, DenseNet, and Inception) are the most popular and have been extensively explored for the classification of TB, as shown in Figure 4. Despite the effectiveness of Deep Learning models in detection and classification tasks, CAD systems for clinical diagnosis are still challenging in a real-world scenario. The physicians and radiologists see CAD intervention as a threat to their jobs rather than a supporting system to improve physicians' performance in terms of time, effort, efficiency, and affordability, especially in developing countries. This review found that most existing works focused on development studies rather than clinical studies.

One of the likely weaknesses of this review is that it is limited to only the studies written in English because there might be other high-quality studies written in other languages. Also, we restricted the computational techniques to only Deep Learning classifiers, which could increase the risk of classifiers bias where studies that employed a hybrid of both Machine Learning and Deep Learning could have achieved better performance. Furthermore, this study did not undertake meta-analyses due to variations of algorithms used. Also, the raw data required to meta-analyze the diagnostic accuracy for most studies were unavailable.

However, it is recommended that studies be carried out using standardized public datasets that contain additional masks of the images that can be used as groundtruth to detect the infected aspect of the pulmonary images. It is highly recommended that models are trained with a set of images and evaluated on a different set of images. For instance, training a model with the Shenzhen datasets and evaluating it on the Montgomery dataset will validate better generalization. It is also recommended that future CAD systems focus more on clinical evaluation and should be able to identify foreign objects such as buttons and rings that look like a nodule on the CXR images, which may lead to misclassification.

## Data Availability Statement

The original contributions presented in the study are included in the article/supplementary material, further inquiries can be directed to the corresponding author/s.

## Author Contributions

All authors listed have made a substantial, direct, and intellectual contribution to the work and approved it for publication.

## Conflict of Interest

The authors declare that the research was conducted in the absence of any commercial or financial relationships that could be construed as a potential conflict of interest.

## Publisher's Note

All claims expressed in this article are solely those of the authors and do not necessarily represent those of their affiliated organizations, or those of the publisher, the editors and the reviewers. Any product that may be evaluated in this article, or claim that may be made by its manufacturer, is not guaranteed or endorsed by the publisher.
